# Redox Control in Acute Lymphoblastic Leukemia: From Physiology to Pathology and Therapeutic Opportunities

**DOI:** 10.3390/cells10051218

**Published:** 2021-05-17

**Authors:** Yongfeng Chen, Jing Li, Zhiqiang Zhao

**Affiliations:** 1 Department of Basic Medical Sciences, Medical College of Taizhou University, Taizhou 318000, China; zhaozhiqiang@tzc.edu.cn; 2 Department of Histology and Embryology, North Sichuan Medical College, Nanchong 637000, China; jingli@nsmc.edu.cn

**Keywords:** acute lymphoblastic leukemia, hematopoietic stem cells, ROS, oxidative stress, pro-oxidative therapy

## Abstract

Acute lymphoblastic leukemia (ALL) is a hematological malignancy originating from B- or T-lymphoid progenitor cells. Recent studies have shown that redox dysregulation caused by overproduction of reactive oxygen species (ROS) has an important role in the development and progression of leukemia. The application of pro-oxidant therapy, which targets redox dysregulation, has achieved satisfactory results in alleviating the conditions of and improving the survival rate for patients with ALL. However, drug resistance and side effects are two major challenges that must be addressed in pro-oxidant therapy. Oxidative stress can activate a variety of antioxidant mechanisms to help leukemia cells escape the damage caused by pro-oxidant drugs and develop drug resistance. Hematopoietic stem cells (HSCs) are extremely sensitive to oxidative stress due to their low levels of differentiation, and the use of pro-oxidant drugs inevitably causes damage to HSCs and may even cause severe bone marrow suppression. In this article, we reviewed research progress regarding the generation and regulation of ROS in normal HSCs and ALL cells as well as the impact of ROS on the biological behavior and fate of cells. An in-depth understanding of the regulatory mechanisms of redox homeostasis in normal and malignant HSCs is conducive to the formulation of rational targeted treatment plans to effectively reduce oxidative damage to normal HSCs while eradicating ALL cells.

## 1. Introduction

Acute lymphoblastic leukemia (ALL) is a type of acute leukemia that mainly manifests as abnormal clonal proliferation of naive or mature T and B lymphocytes and their infiltration of bone marrow (BM), blood, or other organs and tissues, causing BM hematopoietic dysfunction and immune dysfunction. ALL has diverse biological characteristics and substantial clinical heterogeneity. With the continuous discovery of new therapeutic drugs and the ongoing innovation of treatment strategies, the remission rate and survival rate for patients with ALL continue to increase. However, the side effects of drugs and drug resistance in some patients remain problems requiring urgent resolution in clinical ALL treatment.

BM is a major hematopoietic organ. In a hypoxic BM niche, the balance between the production and clearance of reactive oxygen species (ROS) in normal hematopoietic stem cells (HSCs) is critical for the maintenance of normal physiological function. Sufficient evidence indicates that imbalances in redox homeostasis are involved in the development, progression, and relapse of leukemia [1]. Redox dysregulation and increased ROS production are well known and important characteristics of tumor cells, including leukemia cells [2]. In view of redox dysfunction, a weakness of leukemia cells, the implementation of pro-oxidant therapy has created hope for the elimination of leukemia cells. However, increased ROS levels can activate a variety of antioxidant mechanisms in leukemia cells to protect them from oxidative stress injury. In addition, leukemia cells can modify the BM niche into a leukemia growth-permissive and normal hematopoiesis-suppressive leukemia niche, rendering the BM niche a sanctuary for leukemia cells to avoid damage from pro-oxidant agents [3,4,5]. These are important causes of leukemia cells’ resistance to pro-oxidant drugs and ALL relapse.

During the implementation of pro-oxidant therapy for leukemia, the cytotoxic effect of ROS on cells is not selective. Pro-oxidant therapy is akin to a double-edged sword, affecting normal cells while killing tumor cells; for example, it has toxic effects on BM-derived HSCs and can induce side effects such as BM suppression, affecting the therapeutic effects on tumors and even endangering the lives of patients. Therefore, strengthening the cytotoxic effect of ROS on tumor cells while avoiding oxidative damage to normal HSCs has become an important issue in pro-oxidant therapy for leukemia.

## 2. ROS Sources and Effects

ROS are a class of oxygen metabolites that are more active than oxygen and oxygen-containing substances derived from them. The major species of ROS include superoxide, hydrogen peroxide (H_2_O_2_), and hydroxyl radicals [6]. ROS production in the body is the result of the aerobic metabolism of cells. Approximately 2% of the oxygen consumed by aerobic cells is estimated to be diverted to ROS generation [7].

Based on the source, ROS production can be divided into exogenous and endogenous production. Exogenous ROS are formed via stimulation by exogenous factors, such as radiation and drugs. Endogenous ROS are produced by mitochondrial and NADPH oxidase (NOX) pathways [8]. Mitochondria are the main site of ROS production in most eukaryotic cells. During aerobic respiration, most electrons are transported along the respiratory chain and combine with molecular oxygen to form water. However, a small portion of electrons leak out of respiratory chain enzyme complexes I and III, resulting in single-electron reduction in molecular oxygen to form strongly oxidative superoxide anions, which generate hydroxyl radicals and H_2_O_2_ through specific chemical reactions [9]. NOX, which is localized on the cell membrane, is a major source of ROS [10]. External signals, such as bacterial lipopolysaccharide, tumor necrosis factor-α (TNF-α), and interleukin (IL)-1, can stimulate rapid activation of NOX, resulting in a substantial increase in oxygen consumption by cells and a reduction in oxygen molecules to superoxide anions. Superoxide anions are catalyzed by dismutase to generate H_2_O_2_, leading to a rapid increase in ROS levels to eliminate invading pathogenic microorganisms. In addition, the endoplasmic reticulum (ER) and some enzymes, such as lipoxygenase, cyclooxygenase, and xanthine oxidase, can also generate ROS through chemical reactions [11].

Organisms have a complete antioxidant system, which is divided into enzymatic and nonenzymatic antioxidant systems. Enzymatic antioxidant systems include superoxide dismutase (SOD), catalase (CAT), and glutathione peroxidase (GPx). The main function of these antioxidant enzymes is to catalyze the degradation of peroxides. Among them, SOD and CAT are important components of the intracellular antioxidant defense system. SOD includes copper (Cu)/zinc (Zn)-SOD in the cytoplasm and nucleus and manganese (Mn)-SOD in mitochondria. SOD can catalyze superoxide anions to generate H_2_O_2_ and O_2_. The main function of CAT in peroxisomes is to catalyze H_2_O_2_ to generate O_2_ and H_2_O [11]. GPx is an important intracellular enzyme, and in most cases, its activity depends on the micronutrient cofactor selenium. Thus, GPx, which is often referred to as a selenocysteine peroxidase, can decompose H_2_O_2_ into O_2_ and H_2_O. GPx has a critical role in inhibiting lipid peroxidation and therefore protects cells from oxidative stress [12]. Nonenzymatic antioxidants include glutathione (GSH), thioredoxin (Trx), vitamin C, vitamin E, carotenoids, flavonoids, melatonin, and other compounds [13]. These nonenzymatic antioxidants either scavenge free radicals by acting as hydrogen donors to provide hydrogen ions or alleviate oxidative stress by chelating metal ions, trapping free radicals, and neutralizing peroxyl radicals [14,15]. Enzymatic and nonenzymatic antioxidant systems act synergistically to provide comprehensive antioxidant protection for cells and tissues.

Under normal circumstances, intracellular ROS production and clearance remain in dynamic equilibrium. An appropriate ROS level is necessary for the maintenance of normal physiological functions. ROS can regulate cell survival, growth, and differentiation and participate in immune and inflammatory responses. Ultraviolet radiation, pathogen invasion, and inflammation may disrupt the redox state of cells and affect the expression of specific genes through related signaling pathways, resulting in different physiological effects. Redox dysregulation, which is caused by various factors, can lead to overproduction and accumulation of ROS, causing oxidative damage to organelles, proteins, lipids, and DNA and thereby destroying the structural and functional integrity of cells and even causing cell death [16,17].

## 3. Effects of ROS on the Functions of HSCs

HSCs are a group of special cells in hematopoietic tissue with high self-renewal and self-replicating capacities and can differentiate into progenitors of various types of blood cells. HSCs are primarily present in BM. Under normal physiological conditions, approximately 75% of HSCs remain in the quiescent stage (G_0_ phase) of the cell cycle to maintain their self-renewal capacity. In addition, 20% of HSCs are in the G_1_ phase, and less than 5% are active in the cell cycle (defined as the S, G_2_, or M phase) [18,19,20].

Maintenance of normal hematopoiesis requires a dynamic balance among the self-renewal, proliferation, and differentiation of HSCs, and cellular redox homeostasis is an important basis for maintaining this balance [21,22]. HSCs in a hypoxic BM niche mainly rely on anaerobic glycolysis to obtain energy, and the generated ROS level is relatively low. In addition, ROS levels in the BM niche are also lower than those in normal tissues; therefore, HSCs are extremely sensitive to changes in oxidative stress [23,24]. Different ROS levels have different effects on HSCs: low ROS levels are conducive to maintaining HSC quiescence; at moderate levels, ROS act as second messengers, which are involved in regulating the proliferation, differentiation, and mobilization of HSCs; and high ROS levels promote HSCs to leave the quiescent state, damage the self-renewal capacity of cells, induce cell senescence, and may even lead to cell death when oxidative damage is serious. In addition, high levels of ROS are closely related to inadequate host immunity and leukemic transformation [25,26,27].

**Figure 1:**
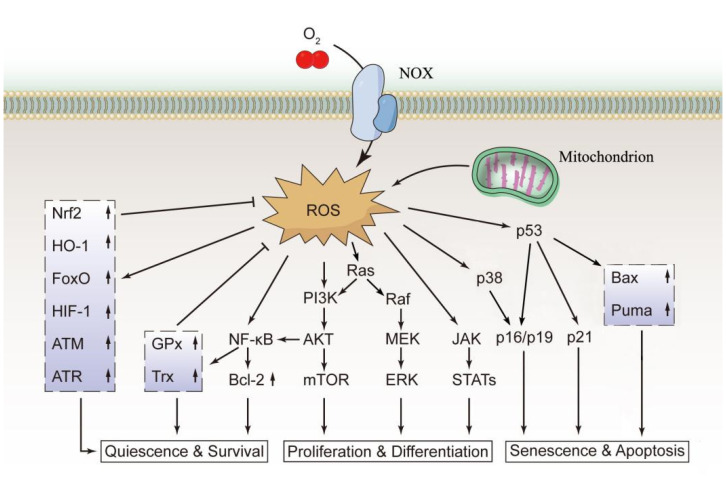
The main redox signaling pathways in normal HSCs. The biological functions of normal HSCs are closely regulated by ROS. ROS can activate the PI3K, MEK, and JAK signaling pathways to promote cell proliferation and differentiation. Excessively high ROS levels activate the p38 or p53 signaling pathway, leading to cell senescence and apoptosis. Additionally, ROS upregulate the expression of Nrf2 and HO-1, which block excessive ROS production through feedback inhibition, thus maintaining intracellular ROS at a normal level to maintain the quiescent state and normal function of the cells. This figure was drawn based on existing research data; its accuracy and more precise signaling mechanisms must be confirmed and supplemented by extensive, in-depth studies.

As active secondary signaling molecules, ROS can activate the nuclear factor kappa B (NF-κB), phosphatidylinositol 3-kinase (PI3K)/protein kinase B (AKT)/mammalian target of rapamycin (mTOR), and mitogen-activated protein kinase (MAPK) signaling pathways to regulate the function of HSCs [28]. NF-κB is an important regulator of both the differentiation and proliferation of HSCs and has a very important role in T and B lymphocyte differentiation and early erythroid proliferation [29]. Nakata et al. showed that the functions of NF-κB in various stages of hematopoiesis are related to its antioxidant and antiapoptotic activities. NF-κB can upregulate the expression of antioxidants, such as GPx and Trx, prevent intracellular accumulation of ROS, and upregulate the expression of the antiapoptotic protein B-cell lymphoma 2 (Bcl-2), thereby protecting HSCs and preventing apoptosis [30]. The PI3K/AKT/mTOR pathway is a representative signaling pathway that regulates a variety of biological activities, including cell growth, differentiation, and proliferation. Proper activation of this pathway can maintain ROS in HSCs at an appropriate level, which is beneficial for the differentiation and proliferation of HSCs. Loss of AKT causes more HSCs to remain quiescent, resulting in severe suppression of hematopoietic cell development [31]. In contrast, constitutive activation of AKT leads to overproliferation of HSCs and subsequent depletion of the stem cell pool [32]. p38 MAPK, a member of the MAPK superfamily, is involved in the regulation of numerous cellular processes, including cell cycle arrest, apoptosis, and senescence [33]. An increase in ROS levels can activate p38 MAPK, and p38 MAPK in turn triggers upregulation of the cyclin-dependent kinase inhibitors p16I^nk4a^ and p19^Arf^, leading to cell cycle arrest and an impaired self-renewal capacity of HSCs [33]. The p53 tumor suppressor protein is a critical regulator of HSC behavior [34]. Many factors, including oxidative stress and DNA damage, can activate the p53 pathway to induce cell cycle arrest, senescence, or apoptosis by upregulating the cell cycle inhibitors p21^Cip1/Waf1^ and p16^Ink4a^ and the proapoptotic proteins p53 upregulated modulator of apoptosis (Puma) and BCL-2-associated X protein (Bax) [35]. The major redox signaling pathways in normal HSCs are shown in Figure 1.

ROS not only directly act on HSCs but also affect the BM hematopoietic niche, thereby regulating the physiological functions of HSCs and determining their fates [36]. Major BM niches include: The endosteal niche, which consists of osteoblasts and their progenitors; the vascular niche, which is composed by BM sinusoidal endothelial cells (BMSECs); and the perivascular niche, where CXC chemokine ligand 12 (CXCL12)-abundant reticular cells (CAR cells) and Nestin^+^ mesenchymal stem cells are present. In addition to cell composition differences, the fundamental difference among these BM niches is the difference in oxygen content. The endosteal niche was believed to be relatively hypoxic, which was thought to promote HSCs’ maintenance of potency. The vascular niche and perivascular niche have relatively high oxygen contents, which might be related to the proliferation, differentiation, and migration of HSCs [2,37]. Cells in the BM niche can regulate HSCs by releasing regulatory signals (e.g., cytokines, chemokines, and adhesion factors), one of the most important of which is stromal cell-derived factor-1 (SDF-1, also known as CXCL12), which binds to the CXCR4 receptor of HSCs and has an important regulatory role in important processes such as the homing, quiescence/proliferation, or migration of HSCs [2]. Recent studies have shown that the CXCR4/CXCL12 axis can counteract HSC exhaustion through selective protection against oxidative stress, and abnormalities in this signaling pathway may be associated with the development of leukemia [2,38]. The settlement of HSCs in the niche relies on the support of N-cadherin, which is a cell adhesion molecule expressed in both HSCs and osteoblasts with a role in the formation of adhesive junctions between HSCs and the BM niche. When the ROS level is upregulated, the expression of N-cadherin in both HSCs and osteoblasts decreases, and N-cadherin-mediated cell adhesion is inhibited, thereby promoting the movement of HSCs out of the BM niche [36,39]. In the hypoxic osteoblastic niche, the plasma membrane surface of quiescent HSCs is highly decorated with signaling molecules such as calcium-sensing receptors and Notch1. Among them, calcium-sensing receptors have a critical role in localizing HSCs to the endosteal surface, and Notch1 interacts with Notch ligands in the osteoblastic niche to maintain HSC quiescence [39]. When the ROS level in HSCs increases, the surface signaling molecules on HSCs are downregulated to different degrees, weakening the interaction between HSCs and the hematopoietic niche and thus affecting the location and quiescence of HSCs in the osteoblastic niche [39].

The function and mechanism of ROS in the maintenance of HSC homeostasis are extremely complex and far from clear. However, it is certain that severe or extended oxidative stress inevitably destroys the homeostasis of HSCs, affecting the self-renewal and differentiation of HSCs and leading to hematopoietic abnormalities. Excessive self-renewal of HSCs may lead to the development of hematologic malignancies, while excessive differentiation of HSCs may induce stem cell pool exhaustion, leading to BM failure [40,41]. Therefore, balanced ROS levels are crucial for maintaining the homeostasis and biological function of HSCs under either normal or stress conditions.

## 4. Redox Regulation in Normal HSCs

ROS generation is inevitably linked with cell respiration [26]. In a hypoxic BM niche, quiescent HSCs have a low energy requirement and mainly rely on glycolysis instead of mitochondrial respiration to obtain energy. Correspondingly, ROS production is relatively low, which is conducive to the maintenance of cell quiescence [42,43]. Piccoli et al. showed that the mitochondrial oxygen consumption rate was low in CD34^+^ HSCs and that extra-mitochondrial oxygen consumption was facilitated by the NOX2 and NOX4 isoforms of the ROS producer plasma membrane NOX with low constitutive activity. Therefore, the authors concluded that the ROS in CD34^+^ HSCs mainly originated from NOX. The ROS released by NOX regulate the biological activity of HSCs through corresponding signal transduction pathways [44].

Because the main role of primitive cells is to provide mature HSCs via proliferation and differentiation, when HSCs proliferate and differentiate, the metabolic state of the cells switches from glycolysis to mitochondrial respiration. During mitochondrial respiration, hematopoietic progenitors produce more adenosine triphosphate (ATP), providing more energy for cell proliferation, differentiation, and motility [26]. Therefore, ROS levels within HSCs change dynamically to achieve compatibility with the biological functions and behaviors of HSCs. During energy metabolism in HSCs, a variety of intracellular and extracellular regulatory mechanisms maintain dynamic equilibrium between the production and clearance of ROS.

In addition to enzymatic and nonenzymatic antioxidant systems, various signaling molecules, including hypoxia inducible factor 1 (HIF-1), ataxia telangiectasia mutation (ATM), forkhead box class O transcription factor (FoxO), AKT, and mTOR, also have important roles in the maintenance of redox homeostasis in HSCs (Figure 1). Among them, HIF-1 is a key factor regulating the cellular hypoxia response. Under hypoxic conditions, HIF-1 regulates the transcription of a variety of hypoxia-responsive genes and participates in hypoxia adaptation, angiogenesis, immune responses, and apoptosis [45,46]. HIF-1 consists of a constitutively expressed subunit (HIF-1β) and an inducible subunit (HIF-1α) that is stabilized under hypoxic conditions. Activation of HIF-1α shifts cellular metabolism to glycolysis rather than mitochondrial respiration, thus limiting ROS generation [2,26]. ATM kinases are key enzymes in the regulation of the stress response to DNA damage. ATM upregulates antioxidant enzymes, negatively regulates ROS production in HSCs, and has an important role in maintaining the self-renewal capacity of HSCs. ATM deficiency inhibits the self-renewal capacity of HSCs [28,47]. FoxO transcription factors are key regulators of HSC antioxidative stress, with FoxO3 having a major role. FoxOs can control the transcription of some ROS regulatory genes and antioxidant enzymes to antagonize ROS, thereby regulating the number, activity, and cell cycle of HSCs [48]. AKT is one of the most common active protein kinases in human cancers. AKT can promote ROS production by increasing oxygen consumption in mitochondria and stimulating oxidative metabolism. Under normal conditions, AKT activity is inhibited by FoxO3 to avoid overproduction of ROS. Overactivation of AKT increases HSC cycling and leads to leukemogenesis [2,26,28]. mTOR is a key regulator of cellular metabolism, and its promoting role in ROS production can be inhibited by tuberous sclerosis complex 1 (Tsc1). Tsc1 knockout in mice leads to mTOR overactivation, enhanced mitochondrial activity, and ROS overproduction in HSCs, thereby disrupting HSC quiescence [49]. Although deletion of the Tsc1 gene does not directly trigger leukemia, Tsc1-knockout mice are prone to developing myeloproliferative disorder, which is a hematological disease with a high risk of progression into acute leukemia [50]. Hence, a complicated signal regulatory network composed of numerous signaling molecules in HSCs subtly regulates the redox balance of HSCs. Abnormalities in any component of the network may interfere with normal hematopoiesis, leading to hematopoietic abnormalities or malignant hematological diseases in serious cases.

The extracellular BM niche has a very important regulatory role in the maintenance of ROS levels in HSCs. The relationship between HSCs and the BM hematopoietic niche is similar to that between seeds and soil: normal hematopoiesis is essential for the extensive proliferation and differentiation of progenitor cells with the support of the hematopoietic niche and the self-maintenance of HSCs [51]. Bone marrow stromal cells (BMSCs) are an important component of the BM niche. BMSCs can secrete various cytokines, such as CXCL12 and stem cell factor (SCF), to regulate the quiescent state of HSCs as well as their proliferation and differentiation, and the CXCL12/CXCR4 axis has the most critical role in the interaction between BMSCs and HSCs [52]. Stromal cells have been proven to have an important role in the maintenance of ROS levels in HSCs. Prostaglandin E2 secreted by α-smooth muscle actin^+^ macrophages has been reported to maintain ROS in HSCs at a low level by inhibiting AKT phosphorylation [53]. In addition, ROS can transfer from HSCs to mesenchymal stromal cells (MSCs) through connexin gap junctions, thereby reducing the ROS level in HSCs [54]. However, with ROS accumulation in stromal cells to a certain extent, stromal cells are also subject to oxidative stress damage, and their supporting effect on HSCs is therefore disrupted, thus affecting the self-renewal capacity of hematopoietic stem/progenitor cells [55], indicating that stromal cells have a limited regulatory effect on the ROS balance in HSCs. Therefore, avoiding exposure to high ROS levels has important significance for HSCs and hematopoiesis-supporting cells and is an important premise for maintaining the normal hematopoietic function of BM.

## 5. The Role of Redox Dyshomeostasis in the Occurrence of ALL

ALL is a highly heterogeneous hematopoietic malignancy whose etiology and pathogenesis are extremely complex and have yet to be fully elucidated. The occurrence of ALL is currently believed to not be caused by a single factor but may be the result of interactions among various factors, including genetics, infection, ionizing radiation, chemical substances, and immune dysfunction, in a complex environment. Clinical data indicate that most patients with ALL harbor acquired genetic alterations that contribute to the increased proliferation, prolonged survival, or impaired differentiation of lymphoid hematopoietic progenitors. Therefore, ALL can be regarded as a type of genetic disease [56].

To date, among the more than 200 reported chromosomal abnormalities in leukemias, balanced translocations are the most common, leading to the generation of fusion genes [57,58]. Abnormal gene expression due to chromosomal translocation or altered function of the encoded fusion protein impairs normal differentiation and yields an aberrant self-renewal capacity, thus having an important role in the malignant transformation of normal hematopoietic stem and progenitor cells (HSPCs). Translocation ETS leukemia-acute myeloid leukemia 1 (*ETV6-RUNX1*) is a chimeric transcription factor that is more common in childhood ALL. The incidence of *ETV6-RUNX1* is considerably higher than that of ALL, suggesting the occurrence of additional genetic events during leukemic transformation after birth [59]. Through the establishment of an *ETV6-RUNX1* transgenic mouse model, Kantner et al. found that although no notable hematopoietic abnormalities were observed in mice, the ROS level in B cells increased, and DNA damage also increased. These results indicated that expression of the oncogene *ETV6-RUNX1* might trigger the second strike by enhancing ROS production, eventually inducing leukemic transformation [59]. Breakpoint cluster region-Abelson (BCR/ABL) is the most common chromosomal genetic abnormality in adult patients with ALL, with a positivity rate of 20–40% [60], and encodes proteins with tyrosine kinase activity, promotes cell proliferation, and inhibits apoptosis [61]. However, the incidence of BCR/ABL-positive samples far exceeds that of the associated leukemias, suggesting that the presence of the BCR/ABL fusion gene alone is insufficient to trigger leukemia [62]. Several recent studies have found that BCR-ABL oncoprotein-expressing cells are associated with a relative increase in intracellular ROS [63]. Studies have shown that BCR/ABL can influence ROS production through manipulation of the NOX complex [64]. In addition, BCR-ABL can also activate the PI3K/AKT/mTOR pathway to promote intracellular ROS production [63]. Compared with normal cells, BCR/ABL-positive cells suffer more oxidative DNA damage (including DNA double-strand breaks) and demonstrate an increased ability to survive DNA damage. BCR/ABL stimulates the efficiency but decreases the fidelity of the repair mechanisms of double-strand breaks, which may be the leading cause of the accumulation of chromosomal aberrations and subsequent malignant lesions [65].

To elucidate the role of ROS in the secondary gene events of ALL, Lim et al. recently investigated factors causing mutations in Janus kinase JAK3, JAK1, and Ikzf3 (encoding Aiolos) using a B-cell acute lymphoblastic leukemia (B-ALL) mouse model [66]. JAKs are nonreceptor tyrosine kinases. Activation of the JAK/signal transducer and activator of transcription (STAT) signaling pathway induces the transcription of genes involved in HSC differentiation and proliferation. [67]. Aiolos belongs to the Ikaros family and is an essential transcription factor for lymphocyte differentiation [68]. Abnormal expression of JAKs and Aiolos has been confirmed to be closely related to the development of ALL [67,69]. Lim et al. found that most mutations with low variant-allele frequency were associated with ROS-induced DNA damage. Application of the JAK inhibitor ruxolitinib can delay leukemia onset, reduce ROS and ROS-induced gene expression signatures, and alter ROS-induced mutational signatures, indicating that JAK mutations can alter the course of leukemia clonal evolution through ROS-induced DNA damage [66]. The clinical biochemical indices of patients with ALL showed a marked increase in the levels of malondialdehyde, an important biochemical index for plasma oxidative stress [70], and a notable increase in the levels of 8-oxodG and 8-OHdG, biomarkers of oxidative DNA damage in urine, in patients with ALL [71,72]. In addition, the level of oxidatively modified DNA bases in lymphocytes from children with ALL was markedly higher than that in children without the disease [73,74,75]. Abundant evidence has shown that ROS have a vital role in secondary gene events in ALL. However, the specific mechanism underlying the development and progression of ALL remains to be further elucidated.

**Figure 2:**
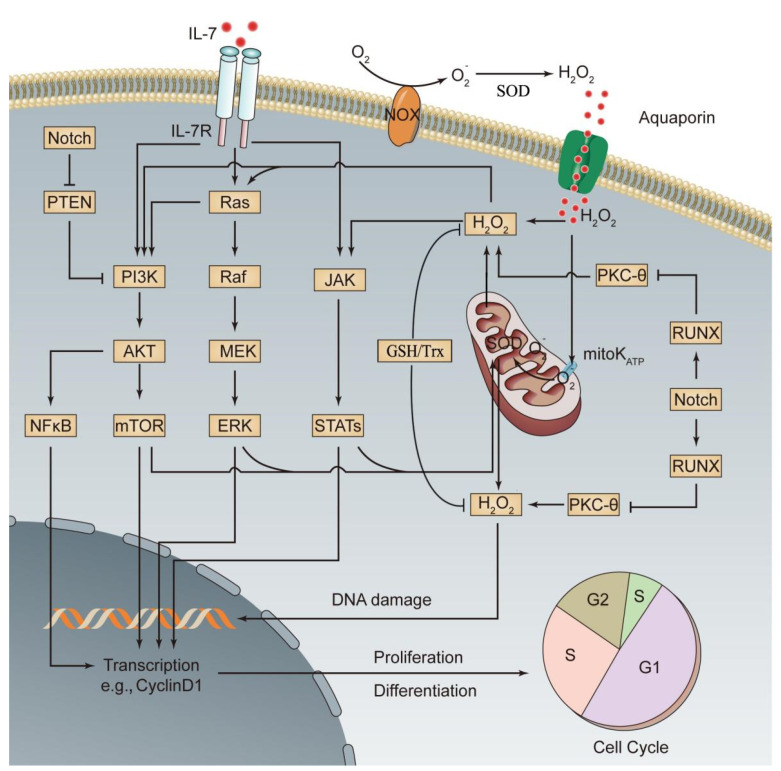
Activated oxidative stress signaling pathways involved in the pathogenesis of ALL. Increases in oncogene-, chemical drug-, or radiation-induced ROS production or abnormal expression of relevant genes leads to activation of three major oxidative stress signaling pathways, PI3K, MEK, and JAK, thus promoting the differentiation and proliferation of leukemia cells. Additionally, activation of oxidative stress signaling pathways promotes mitochondrial ROS production through enhanced oxidative metabolism, which further activates the three oxidative stress signaling pathways, thereby forming a positive feedback signaling pathway. A series of mechanisms in leukemia cells prevent excessive ROS production, thus avoiding cell injury or death (see the text for details). This figure was drawn based on existing research data; its accuracy and more precise signaling mechanisms must be confirmed and supplemented by extensive, in-depth studies. mitoK_ATP_, mitochondrial ATP-sensitive K^+^ channel.

According to the second hit theory, the occurrence of leukemia is the result of the accumulation of multiple gene abnormalities. Abnormal gene expression activates specific signaling pathways to promote the malignant transformation of cells. PI3K/AKT/mTOR, MAPK kinase (MEK)/extracellular signal-regulated kinase (ERK), and JAK/STAT are the major signaling pathways of oxidative stress and are also the representative signaling pathways for abnormal activation of leukemia cells [76,77]. In normal HSCs, overactivation of the above oxidative stress pathways promotes the production and intracellular accumulation of ROS, severely disturbs the normal biological functions of HSCs, and has an important role in leukemia progression [76,77]. Recent studies have shown that abnormal expression of a variety of genes activates the related oxidative stress pathway, thereby affecting ALL progression. These genes mainly include Notch, PTEN, RAS, and IL-7 and its receptor (IL-7R). (1) Notch encodes a transmembrane receptor that regulates normal T cell development. Mutations in this gene are common in T cell acute lymphoblastic leukemia (T-ALL). In T-ALL cells, the withdrawal of Notch signals prevents stimulation of the mTOR pathway by mitogenic factors, indicating that Notch has a positive regulatory role in the mTOR pathway [78]. Mutant Notch can activate the mTOR pathway through the PI3K/AKT pathway or through activation of c-Myc without relying on the PI3K pathway [78,79]. (2) PTEN is a tumor suppressor gene closely related to tumor development. Its encoded protein has dual protein/lipid phosphatase activity and is a major phosphatase with a negative regulatory effect on the PI3K/AKT pathway [80]. Mutation or deactivation of PTEN after translation can result in chronic activation of PI3K/AKT/mTOR signaling in ALL cells, γ-secretase inhibitor (GSI) resistance, and inhibition of p53-mediated apoptosis [79]. (3) RAS is a proto-oncogene, and its encoded proteins are small GTPases, which act as molecular switches. Activated RAS promotes ROS production through NOX stimulation [81] and transduces signals from a variety of cell surface receptors to downstream signaling pathways, such as PI3K/AKT/mTOR and MAPK, to regulate a number of cell fate decisions. RAS-activating mutations and Notch mutations synergistically promote the development and progression of T-ALL [82]. (4) IL-7 and IL-7R are required for normal lymphocyte development. Without them, severe combined immunodeficiency occurs. However, excessive activation of IL-7/IL-7R signaling activates the three oxidative stress signaling pathways, PI3K/AKT/mTOR, MEK/ERK, and JAK/STAT, to promote the development of ALL and the resistance of ALL cells to chemotherapeutic drugs [66,83]. Lim et al. found that ROS induced by IL-7 in the downstream JAK/STAT pathway positively increased JAK/STAT signaling and that the occurrence of B-ALL and the survival of B-ALL cells were dependent on high levels of ROS driven by IL-7-dependent JAK/STAT signaling [66]. Silva et al. reported a positive feedback interaction between the IL-7-mediated PI3K/AKT signaling pathway and ROS. Moreover, activation of this pathway upregulated glucose transport protein type 1 and increased glucose uptake by T-ALL cells. The use of ROS scavengers inhibited the viability of T-ALL cells or even led to cell death [84]. These results suggest that ROS not only drive the development of ALL but are also indispensable for the survival of ALL cells. The major redox signaling pathways associated with ALL transformation are shown in Figure 2, and the corresponding targeted drugs are shown in Table 1.

## 6. Redox Regulation in Leukemia

The hypoxic BM niche is not only a habitat for HSCs but also a natural sanctuary for malignant blood cells. Leukemia cells hidden in the BM niche can avoid the cytotoxicity of chemotherapeutic drugs, resulting in the formation of minimal residual disease. Minimal residual disease is an accepted root cause of drug resistance and relapse in leukemia [96]. A large body of evidence has shown that in a hypoxic environment, the ROS level and its regulation have important roles in leukemia cell survival, proliferation, differentiation, immune escape, and epigenetic changes [97].

Similar to HSCs, primitive leukemia cells also have self-renewal, self-differentiation, and self-proliferation capacities, and their relatively low intracellular ROS levels are conducive to cell survival and stemness maintenance [98,99]. Compared with that in leukemia cells with higher proliferating activity, the ROS level in primitive leukemia cells is lower. This phenomenon has been observed in many types of leukemia cells [2,99]. Giambra et al. also observed that the most aggressive leukemia-initiating cells (LICs) in T-ALL model mice and human T-ALL were characterized by low levels of ROS, and that the increase in ROS levels inhibited the activity of LICs [100], suggesting that the behavior of ALL cells is closely related to ROS levels and oxidative stress status.

Similar to other types of cancer cells, leukemia cells can also undergo glycolysis for energy metabolism. However, a large amount of evidence indicates that leukemia cells mainly gain energy through mitochondrial respiration and that ROS produced through the mitochondrial respiratory chain thus become a major source of ROS in leukemia cells [21,64]. Markedly improved NOX activity has been observed in many leukemia cell lines, including AML, chronic myelogenous leukemia, and promyelocytic leukemia cell lines, indicating that constitutive activation of NOX is another important source of ROS in leukemia cells [101,102,103]. In the in vitro culture of T-ALL cells, the inhibition of complex I of the respiratory chain with rotenone and the disassembly of NADPH subunits with apocynin both abrogated the IL-7-mediated elevation of intracellular ROS levels, confirming that the mitochondrial respiratory chain and NOX are also the main sources of intracellular ROS production in T-ALL cells [84]. Studies have found that in lymphoblastic leukemia cells, xanthine dehydrogenase and xanthine oxidase can oxidize NADH to catalyze the production of ROS, suggesting that in addition to the mitochondrial respiratory chain and NOX complex, certain metabolic/detoxification pathways in lymphoblastic leukemia cells are also important sources of ROS production [104]. In addition, as described above, overactivation of three signaling oxidative stress pathways, PI3K/AKT/mTOR, MEK/ERK, and JAK/STAT, and oncogenes such as BCR/ABL and RAS are related to the changes in redox homeostasis in leukemia cells and increases in ROS levels.

Previous studies have shown that decreased antioxidant defense exists in multiple types of leukemia [105,106,107]. Therefore, redox dysregulation may be one of the causes of the increase in ROS levels in leukemia cells. Sentürker et al. conducted a controlled study with a group of untreated children with newly diagnosed ALL and normal children and confirmed that three antioxidant enzymes, CAT, GPx, and SOD, in blood lymphocytes in children with ALL were lower than those in normal children in the control group [74]. Battisti et al. evaluated the oxidative status and antioxidant defense of patients with ALL and found that CAT and SOD activities in the whole blood of patients with ALL were lower by different levels than those in the normal control group and that SOD activity was lowest in newly diagnosed patients [105]. However, Ben Mahmoud et al. found that the plasmatic activities of CAT and SOD and the plasma levels of reduced GSH were elevated in patients with ALL and that SOD activity and GSH levels were substantially correlated with ALL relapse [108]. Nishiura et al. found that serum levels of Mn-SOD in patients with ALL were substantially higher than those in normal controls, but as the disease subsided, serum levels of Mn-SOD decreased [109]. Although the specific causes leading to large differences in the results between different studies are still unclear, the existing research findings suggest that changes in the antioxidant defense capacity of ALL cells may be related to the development and progression of the disease and the course of treatment. At the onset of leukemia, the decreased antioxidant defense may be related to the occurrence of events such as oxidative DNA damage and genomic instability. During the progression and treatment stages of leukemia, increased antioxidant defense may be an adaptive response to the increase in oxidative stress, enabling leukemia cells to survive under high levels of oxidative stress and to even antagonize the cytotoxic effects of chemotherapy drugs and develop drug resistance.

In the development and progression of leukemia, on the one hand, malignant transformation of HSCs must be initiated through the production of high levels of ROS; on the other hand, continuous intracellular ROS accumulation must be prevented to avoid oxidative damage or cell death, which requires real-time and dynamic regulation of intracellular oxidation and antioxidant status. This regulation process not only depends on the participation of the intracellular antioxidant defense system but also involves a series of other complex intracellular and extracellular mechanisms. The GSH redox system represents one of the most important cellular defense systems against oxidative stress, and high intracellular GSH levels have been linked to treatment resistance in leukemia cells [110]. The Trx system is another major cellular antioxidant network and is composed of NADPH, Trx reductase, and Trx, which can provide electrons for thiol-dependent peroxidases (peroxiredoxins) to remove reactive oxygen and nitrogen species with a fast reaction rate, thereby protecting cells from oxidative damage [1,15]. For relatively quiescent LICs, regulation of the intracellular oxidative stress status is more important for the maintenance of their invasiveness. As mentioned above, Notch is frequently activated by mutations in T-ALL, and activated Notch participates in leukemic transformation by activating the mTOR oxidative stress pathway. However, Notch also downregulates the expression of protein kinase C θ (PKC-θ) through the runt-related transcription factor (RUNX) pathway, thereby inhibiting intracellular ROS accumulation and facilitating the maintenance of LIC activity [100]. In addition, numerous studies have shown that various antioxidant proteins, such as nuclear factor erythroid 2-related factor 2 (Nrf2) and heme oxygenase-1 (HO-1), also have important regulatory roles in the antioxidant responses of ALL cells [111,112].

Extracellularly, ALL cells establish extensive contact with the BM niche via chemokines, adhesion molecules, and exosomes and transform the BM niche into a leukemia growth-permissive and normal hematopoiesis-suppressive leukemia niche to improve the survival of ALL cells in a hypoxic environment [46]. Under oxidative stress conditions, BMSCs produce protective soluble factors, which help ALL cells achieve redox adaptation and reduce oxidative stress damage, ultimately inducing the reversible multidrug resistance phenotype in ALL cells [5]. Tunneling nanotubes (TNTs), which were discovered by Rustom et al. in 2004 using a three-dimensional live cell microscopy, serve as an intercellular communication method [113]. Recent studies have found that ALL cells regulate the BM niche through the use of TNTs to induce the secretion of prosurvival cytokines by signaling to primary MSCs, thereby contributing to the survival of ALL cells [4]. Mitochondria in ALL cells have also been found to be transferred to MSCs through TNTs to alleviate oxidative stress, thereby reducing intracellular ROS levels and avoiding chemotherapeutic drug-induced death [3]. Therefore, the BM niche inhabited by ALL cells has become an important sanctuary for maintaining redox homeostasis and resisting oxidative stress damage. Breaking the antioxidation barrier of leukemia cells is currently an important topic in the promotion of pro-oxidant therapy for leukemia and has received extensive attention from researchers.

## 7. Targeting ROS in ALL Treatment

The redox dysregulation of leukemia cells results in long-term oxidative stress in the cells, and the intracellular ROS levels are higher than those in normal cells. Theoretically, the threshold of ROS tolerance in leukemia cells is also lower than that in normal cells. The ROS concentrations that induce leukemia cell apoptosis may be tolerable to normal cells. Therefore, destroying the redox balance of leukemia cells increases the ROS level to promote apoptosis, which becomes a valuable pathway to eliminate leukemia cells.

Chemotherapeutic drugs commonly used for the treatment of ALL include mitotic inhibitors such as vincristine [114], purine analogs such as cytarabine [3], anthracyclines such as daunorubicin [3] and doxorubicin [115]. These drugs can have antileukemic roles by promoting the production of ROS. Recent studies confirmed that a variety of drugs could disrupt the oxidative balance to promote the death of leukemia cells, including proteasome inhibitors such as bortezomib and marizomib [64,116], histone deacetylase inhibitors (HDACIs) such as vorinostat or entinostat (SNDX-275) [117], Trx inhibitors such as PX-12 [1], and heme oxygenase-1 inhibitors such as protoporphyrin [64]. Thiopurines are the major drug used in the maintenance treatment for ALL. Previous studies have shown that the DNA mismatch repair (MMR) system is a major contributor to thiopurine toxicity. A recent study of a variety of cells, including the MPR-defective human leukemia cell line CCRF-CEM and MMR-defective HeLa-MSH2 cells, showed that 6-thioguanine (6-TG) could be used to induce cell death by promoting intracellular ROS production via an MMR-independent pathway and that the clearance of ROS markedly reduced cell sensitivity to 6-TG, suggesting that pro-oxidant stress might be an important mechanism by which 6-TG exerts its cytotoxic effect [118]. Results obtained by multiple research groups also showed that increased oxidative stress might be a mechanism involved in thiopurine-induced cytotoxicity [119,120].

Mitochondria are the main production site for intracellular ROS. Therefore, targeting mitochondria is a suitable strategy to disrupt cell redox homeostasis, induce oxidative stress, and promote the apoptosis of leukemia cells. Many mitochondrial inhibitors that can promote ROS production are undergoing clinical trials for leukemia treatment. Metformin, an antidiabetic drug, has been confirmed to inhibit the mitochondrial production of adenosine triphosphate and increase ROS [121]. The combined use of metformin and rotenone substantially increased the drug sensitivity of Jurkat (an ALL cell line) cells [122]. Tigecycline, an FDA-approved antibiotic, is an attractive candidate for ALL treatment. Recent studies have found that tigecycline triggers an energy crisis and oxidative stress and damage by inhibiting mitochondrial respiration in ALL cells [123]. Fu et al. have found that tigecycline has a certain selective cytotoxic effect on ALL cells, while its toxicity to normal HSCs is relatively small. Further analysis showed that the enhanced mitochondrial biogenesis and increased oxygen consumption rate in ALL versus normal HSCs might be responsible for the different sensitivities of the two types of cells to tigecycline [123]. Adaphostin is a tyrphostin that was originally intended to inhibit BCR/ABL kinase by competing with peptide substrates [124]. Studies have found that adaphostin can also increase ROS levels through the inhibition of mitochondrial respiration, thereby exerting an anticancer effect [2,125]. The ROS produced by adaphostin can overcome even the most potent imatinib resistance in chronic myelogenous leukemia and ALL [124].

During pro-oxidant therapy for leukemia, drug resistance caused by redox adaptation in leukemia cells is a complicated issue that severely affects the efficacy of leukemia treatment. In recent years, numerous studies have shown that targeting the antioxidative defense of leukemia cells can not only increase the sensitivity of leukemia cells to pro-oxidant drugs and promote the ROS-induced apoptosis of leukemia cells but also reduce the side effects of chemotherapeutic drugs to normal cells (see next section for details) [126,127,128]. In addition, it is also a daunting and interesting strategy to cut off the connection between leukemia cells and other cells in the BM niche and to remove the protection provided by the BM niche for leukemia cells. Based on the current understanding of the interaction between the BM niche and leukemia cells, drugs targeting potential therapeutic targets, such as TNTs, signal transduction pathways, cytokines/chemokines and their receptors, and adhesion molecules, have been developed and achieved fruitful results in in vitro and animal experiments. According to a report by Burt et al., the inhibition of TNTs markedly reduced the protection of ALL cells by MSCs, thereby promoting the apoptosis and death of ALL targets induced by two ROS-inducing chemotherapy agents, cytarabine and daunorubicin [3]. Chiarini et al. showed that the inhibition of the CXCL12/CXCR4 interaction promoted the detachment of leukemia cells from their protective BM niches, thus making them more sensitive to chemotherapeutic drugs [129]. However, one of the potentially serious drawbacks of inhibiting the CXCL12/CXCR4 interaction is the release of a large number of leukemia cells into the peripheral blood, which increases the potential of leukemia cells to infiltrate extramedullary organs. Therefore, all current open clinical trials using CXCR4 inhibitors as chemosensitizers combine these inhibitors with a variety of chemotherapeutic drugs to increase the possibility of killing activated leukemia cells.

Notably, ROS not only have an important role in the induction of apoptosis but are also strong inducers of autophagy [130,131,132]. Pro-oxidant drugs such as vorinostat and vincristine induce autophagy while increasing the ROS levels in ALL cells and inducing apoptosis. Blocking autophagy markedly increases the cytotoxicity of pro-oxidant drugs, confirming that autophagy is an important protective mechanism of ALL cells in pro-oxidant therapy [133,134]. Therefore, the effect of ROS-mediated autophagy cannot be ignored in the development of drug resistance in leukemia cells while killing leukemia cells through ROS-mediated apoptosis. Cutting off the ROS-mediated autophagy pathway is necessary for improving the efficacy of pro-oxidant therapy.

## 8. Impact of Pro-Oxidant Therapy on Normal HSCs

In addition to the drug resistance caused by redox adaptation, another thorny issue that has been affecting the efficacy of pro-oxidant therapy for leukemia is the side effects of pro-oxidant drugs on normal cells. The oxidative damage of ROS to cells is not selective. Pro-oxidant drugs are a double-edged sword, inevitably causing oxidative damage to cells in normal tissues while killing tumor cells, resulting in serious side effects. HSCs in a hypoxic BM niche have low levels of differentiation, highly active division, relatively low levels of intracellular ROS, and high sensitivity to oxidative stress. Therefore, compared with cells in other tissues, HSCs are more vulnerable to the damage caused by pro-oxidant drugs [135]. In addition, persistent oxidative DNA damage can lead to senescence and loss of the self-renewal capacity of HSCs, resulting in long-term BM suppression and hematopoietic failure in patients [136,137].

Researchers have conducted extensive research on the adverse effects of pro-oxidant therapy on BM hematopoiesis. Based on existing research results, targeting the antioxidative defense of leukemia cells could be a beneficial strategy for patients with ALL. The GSH/GPx system is a major regulator of cellular redox homeostasis. Therefore, theoretically, its impairment may induce severe oxidative stress in cells [2]. Schoeneberger et al. confirmed that depleting GSH with buthionine sulfoximine (BSO) to inhibit the antioxidative defense of ALL cells can induce ROS production and promote the apoptosis of ALL cells under the action of a Smac mimetic, BV6. In contrast, BSO treatment did not make nonmalignant lymphohematopoietic cells from healthy donors sensitive to BV6, suggesting that BSO has a certain tumor selectivity [110]. Preclinical studies showed that SOD inhibitors, such as 2-methoxyestradiol [138] and ATN-224 [127], have antileukemic abilities and that 2-methoxyestradiol has high selectivity and kills ALL cells in a targeted way without affecting normal hematopoietic stem/progenitor cells [128]. Fidyt et al. found that auranofin, an inhibitor of antioxidant enzymes in the Trx system, has high selectivity for B-cell precursor ALL cells [139].

The weak selectivity of chemotherapeutic drugs for leukemia cells is an important cause for severe side effects. In the past decade, the tumor-specific prodrug strategy developed based on the high levels of ROS in tumor cells has provided a novel approach to improve the selectivity, enhance the efficacy, and reduce the side effects of chemotherapy. ROS-responsive anticancer prodrugs, which are designed based on ROS-sensitive linkers, are composed of anticancer drugs, ROS-sensitive linkers, and other functional units. They are designed to mask the original cytotoxic activity of drugs and can respond to high-level tumor-specific ROS in tumor sites to trigger the breakage of sensitive linkers to release active drugs, ultimately achieving the goal of selectively killing tumor cells [140,141,142]. Sensitive linkers currently available for the design of ROS-responsive prodrugs include aryl boronic acid or ester, alkyl thioether or selenide, thioketal, peroxalate ester, and aminoacrylate [140] (Table 2). Aryl boronic acid and ester have been the main focuses of research on ROS-responsive prodrugs for leukemia treatment. Since 2011, the research group led by Peng has performed much research on using aryl boronic acid or ester to construct ROS-responsive nitrogen mustard anticancer prodrugs. Nitrogen mustard is one of the earliest drugs for the treatment of malignant tumors and has an antitumor role by increasing ROS levels in tumor cells [143,144]. Peng et al. covalently connected boronic acid and nitrogen mustard to form an ROS-activated nitrogen mustard prodrug and confirmed, through in vitro experiments, that the drug markedly improved the toxicity to leukemia cells that have inherently high levels of ROS [145]. Recently, Liao et al. designed and constructed an HDACI prodrug by utilizing the sensitivity of aryl boronic acid to ROS; aryl boronic acid can be activated by high levels of ROS in AML cells to release cytotoxic HDACIs, thereby achieving precise and highly efficient antileukemic effects [146]. The research group led by Mokhir designed the so-called aryl boronic ester aminoferrocene dual prodrugs, which can simultaneously induce catalytic ROS generation and inhibit the antioxidant system of cells. The efficacy of the dual prodrugs in a variety of leukemias was considerably improved as evidenced by in vivo and in vitro experiments, and no toxicity to normal cells was observed [147,148,149,150]. Although great progress has been made in ROS-responsive anticancer prodrugs, there are still many issues. For example, quiescent LSCs have low levels of intracellular ROS that may not be sufficient to activate the prodrug system. Therefore, researchers attach great importance to the compensation strategy of adding ROS promoters (β-lapa, glucose oxidase, photosensitizers, and ascorbic acid) into the ROS prodrug system to provide additional ROS [140]. However, this ROS compensation strategy should be selective; otherwise, it will promote the generation of ROS in HSCs and lead to unexpected injury.

Natural medicines have received increasing attention in reducing cytotoxicity and improving the efficacy of chemotherapy. Many antitumor natural drugs, such as curcumin, resveratrol, parthenolide, and catechins, are also closely related to ROS and the induction of tumor cell apoptosis. They can replace traditional chemotherapeutic drugs to some extent or reduce the doses of traditional chemotherapeutic drugs to reduce side effects. Curcumin induces Jurkat cell apoptosis through the induction of intracellular ROS production, loss of mitochondrial membrane potential, and depletion of GSH, without noticeably affecting normal cells [156]. Resveratrol synergizes with barasertib and everolimus to enhance the cytotoxicity of these chemotherapeutic drugs and induce the death of Jurkat cells by promoting the production of ROS, without affecting normal cells [157]. Similarly, parthenolide [158] and piperlongumine [159] also effectively reduce the side effects and improve the efficacy of leukemia treatment, suggesting that natural antitumor drugs have excellent application prospects for the treatment of leukemia and protection of BM hematopoiesis (Table 3).

## 9. Conclusions

In summary, as active secondary signaling molecules, ROS have important inductive and regulatory roles at various stages of ALL development and progression. Studies have shown that external factors such as ionizing radiation, chemicals, and drugs, internal factors including the occurrence of fusion genes such as *ETV6-RUNX1* and *BCRABL*, activation of oxidative stress signaling pathways, and defects in antioxidant defense systems might all be able to promote the production and intracellular accumulation of ROS, which may seriously disrupt the normal biological functions of hematopoietic cells and may induce genetic lesions considered determinant and crucial for leukemic transformation of normal HSCs and/or hematopoietic progenitors, ultimately leading to the development of leukemia. The mechanism of action of ROS on proteins and lipids at the molecular level is basically clear. However, many topics relevant to the development and progression of ALL require further study, including the dynamic changes in the levels of intracellular ROS and various antioxidants, how ROS activate oncogenes at the nucleic acid level, and the specific mechanism of action of ROS in subsequent signal transduction. An inadequate understanding of redox signaling in normal and malignant HSCs severely limits the efficacy of ALL treatment. To date, drug resistance and side effects caused by pro-oxidant drugs remain an urgent and difficult problem in the treatment of leukemia. Deepening the understanding of redox signaling in physiological and pathological conditions and the continuous development of new biological techniques and materials will help to identify more effective therapeutic targets and develop specific targeted drugs, thereby substantially promoting the precise diagnosis and treatment of ALL and improving the therapeutic effect for ALL on the basis of providing practical protection of BM-derived HSCs.

## Figures and Tables

**Table 1:** ALL redox signaling pathway targets and representative drugs.

Signaling Pathway	Targets	Representative Drugs	Antileukemic Effect	Refs
BCR/ABL	Tyrosine kinase inhibitor (TKI)	Imatinib	First-generation TKI that can block the ATP-binding sites of BCR-ABL and prevent activation of the conformation of oncogenic proteins	[85]
		Nilotinib	Second-generation TKI and high-affinity aminopyrimidine-based ATP-competitive inhibitor with more specific inhibition of BCR/ABL activity	[86]
		Dasatinib	Second-generation TKI that can bind to inactive and active BCR/ABL kinase and inhibit Src family kinases and c-Kit	[86]
		Bosutinib	Third-generation TKI and potent dual inhibitor of Src and ABL kinases with longer-term safety than second-generation and other third-generation TKIs	[87]
		Ponatinib	Third-generation TKI that is effective for known mutations in imatinib-resistant genes (including T315I)	[88]
Notch	γ-secretase inhibitor (GSIs)	BMS-906024	Inhibits the activity of Notch signaling by downregulating the expression of multiple known target genes of Notch but has no marked effect on c-Myc	[89]
		PF-03084014	Downregulates the level of the Notch intracellular domain and the expression of Notch target genes Hes-1 and c-Myc and induces cell cycle arrest and apoptosis of T-ALL cells	[90]
PI3K/AKT/mTOR	PI3K-δ inhibitor	Idelalisib	Downregulates the level of AKT phosphorylation in B-ALL cells, inhibits cell proliferation, and blocks the homing of B-ALL cells into the bone marrow	[91]
		NVP-BKM120	Downregulates the phosphorylation levels of AKT and mTOR in T-ALL cells, inhibits cell cycle progression, and promotes apoptosis	[92]
	AKT inhibitor	MK-2206	Downregulates AKT phosphorylation levels in both T-ALL and B-ALL cell lines (it can also promote PTEN phosphorylation in B-ALL cell lines), inhibits cell proliferation, and promotes apoptosis	[93]
	PI3K/mTOR inhibitor	PI-103	More potent than inhibitors that are selective only for PI3K or for mTOR and can effectively induce cell cycle arrest and apoptosis in T-ALL cells	[94]
JAK/STAT	JAK inhibitor	Ruxolitinib	JAK1/2 inhibitor that can reduce ROS and ROS-induced gene expression signatures and inhibit the growth of leukemia cells	[66]
RAS	MEK inhibitor	Selumetinib Trametinib MEK162	Reduce ERK phosphorylation and induce apoptosis in the RAS-mutant MLL-rearranged ALL cells	[95]

**Table 2:** Common ROS-responsive linkers.

ROS-Responsive Linker	Activation and Active Drug Release Mechanisms under the Action of ROS	Refs
Alkyl thioether/selenide	In the presence of oxidative conditions, thioether-containing polymers undergo phase transition from hydrophobic sulfide to more hydrophilic sulfoxide or sulfone. The increased hydrophilicity promotes the hydrolysis of ester bonds, thereby accelerating drug release.	[140,151]
Aminoacrylate	Prodrugs formed from aminoacrylate, in which electron-rich alkenes are easily oxidized by ROS, undergo [2 + 2] cycloaddition reaction, thereby releasing molecular drugs via self-breakage.	[140,142]
Anthocyanin	Under oxidative stress, anthocyanins can undergo responsive breakage to release drugs.	[152,153]
Arylboronic acid/ester	B—C bonds are oxidized through coordination of ROS with boron atoms to form borate and arylphenols. The drugs are released through the self-breakage of arylphenols.	[142]
Ferrocene	Ferrocene has certain hydrophobicity and can form water-soluble salts after oxidation. When ferrocene is attached to the hydrophobic end of a copolymer, ROS-responsive drug release can be achieved.	[153,154]
Peroxalate ester	Peroxalate ester can be easily oxidized by ROS to generate the intermediate dioxetanedione, which is rapidly decomposed into carbon dioxide, and release drugs.	[151,155]
Poly(propylene sulfide) (PPS)	Under the oxidation of ROS, the sulfur in propylene sulfide is oxidized to form sulfur oxides, resulting in increased hydrophilicity and promoting drug release.	[153]
Thioketal	Thioketal can be rapidly cleaved by ROS species and degraded into acetone and thiols as byproducts to achieve drug release.	[151]

**Table 3:** Natural compounds that exert anti-ALL effects through pro-oxidation.

Natural Compound	Cell Type	Action	Possible Anti-Leukemia Mechanism	Refs
Ardisiacrispin B	CCRF-CEM human T-cell ALL cell line	Induces apoptosis	Activates caspases 8 and 9 and caspase 3/7 and increases ROS production	[160]
Artesunate	Jurkat, CEM, and Molt-4 human T-cell ALL cell lines	Induces apoptosis	ROS-dependent mitochondria-mediated pathway	[161]
Baicalin	Jurkat human T-cell ALL cell line, human peripheral blood mononuclear cells (PBMCs) isolated from blood of healthy volunteers	Induces apoptosis in Jurkat cells while having little cytotoxicity on PBMCs	ROS-dependent mitochondria-mediated apoptotic pathway	[162]
Camalexin	Jurkat human T-cell ALL cell line, human lymphoblasts, and primary fibroblasts	In the micromolar range, camalexin exhibits time- and concentration-dependent cytotoxicity to Jurkat cells but has little cytotoxicity to normal cells.	ROS-dependent mitochondria-mediated apoptotic pathway	[163]
Curcumin	697, REH, RS4;11, and SupB15 human B-cell precursor ALL cell lines	Induces apoptosis	ROS-dependent mitochondria-mediated intrinsic pathway	[164]
Cyanidin-3-rutinoside	HL-60 human promyelocytic leukemia cell line, CCRF-CEM and Molt-4 human T-cell ALL cell lines, human PBMCs isolated from healthy donors	Induces apoptosis in leukemia cell lines while having little cytotoxicity to normal PBMCs	ROS-dependent activation of p38 MAPK and JNK	[165]
Matrine	Human ALL B-lymphocytes	Promotes apoptosis in ALL cells	Promotes ROS generation, upregulates Bax, and downregulates Bcl-2	[166]
Parthenolide	SEM and RS4;11 pre-B ALL cell lines, human T-cell ALL cells	Induces rapid apoptotic cell death	Promotes ROS generation	[158,167]
Piperlongumine	GC-resistant B-ALL cell lines and GC-sensitive B-ALL cell lines, human PBMCs B cells	Regardless of GC-resistance, piperlongumine inhibits the proliferation of all B-ALL cell lines but not normal B cells and induces apoptosis	Promotes ROS generation	[159]
Resveratrol	Jurkat human T-cell ALL cell line, normal lymphocytes	Resveratrol synergizes with barasertib or everolimus to enhance the cytotoxic effect on ALL cells without affecting normal lymphocytes	Promotes ROS generation	[157]
Sanguinarine	697, REH, RS4;11, and SupB15 human B-cell precursor ALL cell lines	Promotes ALL cell apoptosis	Promotes ROS generation, upregulates Bax, and downregulates Bcl-2	[168]
Triptolide	ALL cell line (NALM-6/R) with cross-resistance to cytarabine (araC) and doxorubicin (ADM)	Reversal of the drug resistance of ALL cells inhibits cell proliferation, induces apoptosis in vitro, and inhibits tumor growth in a mouse xenograft model	Impairs mitochondrial membrane potential and increases ROS production	[169]
